# On the Treatment of Hematemesis and Melæna

**Published:** 1850-10

**Authors:** J. M. Neligan

**Affiliations:** Physician to Jervis Street Hospital, Dublin


*On the Treatment of Hematemesis and Melœna.* By Dr. J. M. NELIGAN, Physician to Jervis Street Hospital, Dublin.—[Dr. Neligan remarks that the greater danger of hematemesis and the greater difficulty of controlling it, as compared with melœna, seem to depend on the size of the stomach permitting a large accumulation of blood in its cavity before it contracts; its contraction "being the natural mechanical method by which the bleeding is checked." Thus, he observes:]

In the administration of remedies to stop the hemorrhage, the chief indication to be fulfilled is therefore manifestly to produce contraction of the stomach, whether the bleeding be symptomatic of organic disease or not. With this view we should be guided in the choice of styptics or astringents, and they should be administered in a concentrated form, or rather as little diluted as possible, so as not to distend the stomach. In hematemesis or melœna, I place most reliance on the oil of turpentine as a styptic, and on gallic acid as an astringent; the former, by its stimulant property, excites the stomach and intestines to contract, and the latter may be given in the solid form, and is very powerful in small doses. Ice has often been prescribed to check hemorrhage from the stomach; but its tendency, when dissolved, is to distend the organ, and to favor further bleeding by diluting the blood that may have previously escaped from the vessels, constitutes, I think, a very serious objection to its employment. As to blood-letting, it is only admissible in those few cases where the hemorrhage seems to be dependent on general plethora, and even in these must be regarded rather in the light of after-treatment than of an immediate remedy.

The diet in all cases of hemorrhage from the stomach or intestines must be absolute. Nourishment should be given in a concentrated liquid form, perfectly cold, and in the smallest quantities at a time; and a return to the usual articles of diet should be permitted with the utmost caution.

I regard gallic acid as our most valuable astringent in hemorrhage from the mucous membrane of the digestive organs, from the uterus —in many cases of bleeding from which I have seen it prove of the greatest service, and from the urinary organs.—*Dub. Jour., May* 1850, *p.* 348.

